# Forecasting blood demand for different blood groups in Shiraz using auto regressive integrated moving average (ARIMA) and artificial neural network (ANN) and a hybrid approaches

**DOI:** 10.1038/s41598-022-26461-y

**Published:** 2022-12-20

**Authors:** Seddigheh Edalat Sarvestani, Nahid Hatam, Mozhgan Seif, Leila Kasraian, Fazilat Sharifi Lari, Mohsen Bayati

**Affiliations:** 1 Student Research Committee, School of Health Management and Information Sciences, Shiraz University of Medical Sciences, Shiraz, Iran; 2 Health Human Resources Research Center, School of Health Management and Information Sciences, Shiraz University of Medical Sciences, Almas Building, Alley 29, Qasrodasht Ave, Shiraz, 71336-54361 Iran; 3 Department of Epidemiology, School of Health, Non-Communicable Diseases Research Center, Shiraz University of Medical Sciences, Shiraz, Iran; 4 Blood Transfusion Research Center, High Institute for Research and Education in Transfusion Medicine, Tehran, Iran; 5 Shiraz Blood Transfusion Center, Shiraz, Iran

**Keywords:** Health policy, Health care economics

## Abstract

Providing fresh blood to keep people in need of blood alive, has always been a main issues of health systems. Right policy-making in this area requires accurate forecasting of blood demand. The current study aimed at predicting demand for different blood groups in Shiraz using Auto Regressive Integrated Moving Average (ARIMA), Artificial Neural Network (ANN) and a hybrid approaches. In the current time series analysis, monthly data of the Shiraz hospitals and medical centers demand for 8 blood groups during 2012–2019 were gathered from Shiraz branch of Iranian Blood Transfusion Organization. ARIMA, ANN and a hybrid model of them was used for prediction. To validate and comprise ARIMA and ANN models, Mean Square Error (MSE) and Mean Absolute Error (MAE) criteria were used. Finally, ARIMA, ANN and hybrid model estimates were compared to actual data for the last 12 months. R3.6.3 were used for statistical analysis. Based on the MSE and MAE of models, ARIMA had the best prediction for demand of all blood groups except O+ and O−. Moreover, for most blood groups, ARIMA had closer prediction to actual data. The demand for four blood groups (mostly negative groups) was increasing and the demand for other four blood groups (mostly positive ones) was decreasing. All three approaches including ARIMA, ANN and the hybrid of them predicted an almost downward trend for the total blood demand. Differences in the performance of various models could be due to the reasons such as different forecast horizons, daily/month/annual data, different sample sizes, types of demand variables and the transformation applied on them, and finally different blood demand behaviors in communities. Advances in surgical techniques, fetal screening, reduction of accidents leading to heavy bleeding, and the modified pattern of blood request for surgeries appeared to have been effective in reducing the demand trend in the current study. However, a longer time period would certainly provide more accurate estimates.

## Introduction

Blood is a vital factor in the human body that transports nutrients and oxygen to all other cells. If some blood is lost for different reasons, it should be replaced by transfusing blood and its products in proportion to the volume lost, because insufficient blood causes the body to have irreversible functional defects^1–3^. Therefore, availability of blood is important for medical, military, and emergency situations^4^. Factors such as the lack of accurate and suitable alternatives to human blood, population growth, and aging have increased the need for more blood. On the other hand, blood products cannot be stored for a long time due to high spoilage risk^5^.

Providing healthy blood in normal or critical situations is an issue that health systems have always faced^2^. Ensuring the availability of enough blood and blood components and responding blood demand in a way that reduces wastage and spoilage is important because failure to meet the demand for blood and blood products can lead to increased mortality and impose costs on the community^6^. A prerequisite for adequate blood provision to the population is to balance the number of blood donations with the demand for blood^7^. Thus, predicting the level of demand for blood can provide evidence to decide on preventing imbalances between supply and demand in the future^8,9^.

It seems that predicting the demand for blood in order to respond to it in the future is necessary for three main reasons, first of which is the increased demand due to the increase in burden of diseases, the development of treatment methods, increased level of health status, and the increase in wars and natural disasters in recent years. The second reason is the increase in blood supply costs due to safety testing and cross-matching, and the third is the age difference between blood donors and applicants. In other words, the aging of current donors over time will result in reduction of blood donors and imbalance between blood demand and supply will occur^10,11^.

The World Health Organization (WHO) has reported the need for 5 to 15 units of blood per active hospital bed^12^. In Iran, a study conducted in Kerman indicated that blood demand in university hospitals, social security hospitals, and private hospitals were 15.09, 6.1, and 5.2 units per active bed, respectively^13^. Thus, making required predictions in order to meet this demand, especially in times of crisis, seems necessary.

Various studies have used different methods to predict blood demand, the most valid and popular of which are the Artificial Neural Network (ANN) and the Auto Regressive Integrated Moving Average (ARIMA). The former has the following characteristics: intelligence, high data processing speed, adaptability to environmental changes, ability to be learned and taught, and nonlinear modeling capability. The latter is a linear combination based on past errors and past values of a static series and includes changing trends, random interference and periodic changes, and lack of changing other related random variables during time series analysis^14,15^. Moreover, hybrid approaches are suggested for time series forecasting^16^.

Few studies have been conducted in this field, mostly in developed countries. For example, Turkulainen introduced the ARIMA with exogenous regressors (ARIMAX) model as the leading model for predicting blood demand in Finland^17^. Fortsch and Khapalova in the United States used daily data and six different methods, and introduced Autoregressive Moving Average (ARMA) as the best model for predicting the total demand and the demand for all blood groups except A−^18^. Pereira showed that in one-year time horizons, red blood cell (RBC) demand forecasting through ARIMA or Exponential Smoothing Models (ETS) methods performed better while ETS had better performance than other forecasting methods for longer time horizons^19^.

Given that few studies have been conducted in this field in developing countries, especially Iran, and since different studies have provided different results, the present study aimed to compare ANN and ARIMA methods for predicting the demand for different blood groups and identify blood demand trends in Shiraz.

Shiraz is one of the metropolises of Iran with a population of nearly 1869,000^20^. In addition, it has major centers for providing important and sub-specialized medical services in the south of the country, so that patients from neighboring provinces and the Persian Gulf countries come to Shiraz to receive services.

## Methods

In order to predict the demand for blood groups using ANN and ARIMA in the present study, monthly data related to the demand of Shiraz hospitals and medical centers for 8 blood groups (A+, A−, B+, B−, AB+, AB−, O+, O−) during 2012–2019 were collected from Shiraz branch of Iranian Blood Transfusion Organization using a researcher-made form.

Before estimating any model, the normality of the data should be ensured. In this study, the Shapiro–Wilk test was used to check data normality. The Box–Cox transformation and, in some cases, Log transformation were also used for normalization.

### ARIMA model

An ARIMA model is a linear combination of a past observation of stationary series and error terms. It is usually presented by ARIMA(p,d,q)(P,D,Q), in which p, q, d, P, Q, and D showes autoregressive order, moving average order, number of difference, seasonal autoregressive order, seasonal moving average order, and number of seasonal difference, respectively. Autocorrelation (ACF) and Partial Autocorrelation (PACF) graphs were used for checking Autocorrelation and seasonal changes of data. Final models were selected using Akaike information criterion (AIC), corrected Akaike Information Criterion (AICc), and minimum Bayesian information criterion (BIC)^15^.

Finally, White neural network test, McLeod–Li test, Box–Ljung test, and Shapiro–Wilk test were applied to check stationarity, heteroscedasticity, independence, and normality of model residuals, respectively.

### ANN model

ANN includes artificial neuron, proccessing connected nods, can indicate complex behavior of connection between proccessing components and components parameter. Several row neurons constitue a layer, and several layers constitue a network.

**Figure 1:**
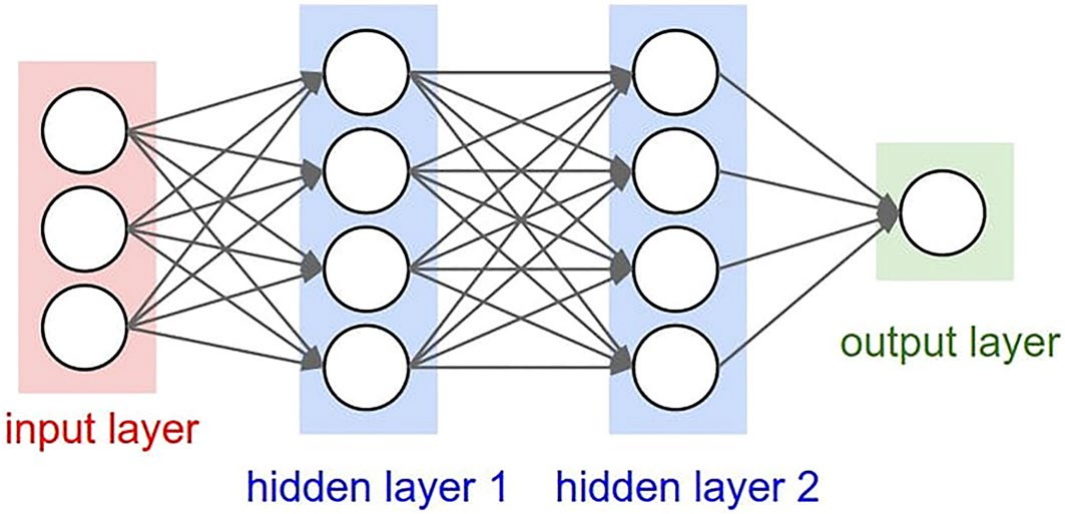
Structure of an artificial neural network.

In the current study ANN Multi-Layer Perceptron (MLP) with Back-Propagation learning algorithm was used. This ANN is constructed from three general layers including input, hidden and output (Fig. 1). Input layer is formed from past time series information of demand. Output layer predict future demand. Hidden layers includes several neurons that selected according to minimum mean square error (MSE)^14^. In other words, a g reedy search algorithm, based on MSE criterion, was used to determine the optimal number of hidden layers and number of neurons in each hidden layer. The applied activation function for prediction in each neuron, is as following:

$${\widehat{y}}_{t}={\widehat{\beta }}_{0}+\sum_{j=1}^{k}{\widehat{\beta }}_{j} \psi \left({x}_{t}^{\mathrm{^{\prime}}}{.\widehat{\gamma }}_{j}\right),$$

Where ${x}_{t}$ is constituted from p steps of ${y}_{t}$ observation. Moreover, $\psi$ function in logarithmic from, is given in the following equation:

$$\psi \left({x}_{t}^{^{\prime}}{\widehat{\gamma }}_{j}\right)={\left[1+\mathrm{exp}\left(-{\widehat{\gamma }}_{j0}+\sum_{i=1}^{p}{\widehat{\gamma }}_{ji}.{y}_{t-1}\right)\right]}^{-1} \quad j=1,\dots ,k$$

In the above neural network with a hidden layer, logit function provides a non-linear fit. K represents the nodes in the hidden layer^21^. It is worth mentioning that the activation function in this type of applied ANN was a combination of logit link and autoregressive models.

Network learning has been done in such a way that initially almost 37% of the data (35 time points) were randomly selected and held out as validation set. The size of this validation set was proportional to exp(−1). The remaining points were considered as train set. Train set was used for training the network; however the coefficients and weights were estimated in such a way that the resulted MSE of predicted value for validation set and also the observed value from this set, be minimized. In other words in order to prevent over fitting the MSE of validation set was used as the convergence criterion. The convergence of the network was defined as not decreasing the MSE from a certain predefined value) i.e. 0.0001). If the algorithm has not converged after a thousand of iterations, the search is stopped. This network learning was repeated 100 times for each blood group and the final prediction was the averaged prediction of these 100 converged networks.

The prediction was in the way that the k recent lags were applied to predict the (k + 1)th one; and this sequence were continued to complete the prediction set. The number of required lags for different blood groups were also determined thorough greedy searches.

As a summary, the combination of logit link and autoregressive models was used as activation function. The selected algorithm is consistent with the time series data used in current study. Moreover, sufficiency of fitted models can be confirmed by residual assessment (see results section).

### The hybrid model of ARIMA and ANN

Actually, ARIMA models is the most common traditional method for time series data modeling and forecasting^16,22–24^. On the other hand, it has been shown in other studies that the ANN algorithm has a better fit than many traditional methods for prediction in non-linear data and data with outlier observations^16,25,26^. Moreover, introducing only one method as the best approach for all conditions is impossible and even illogical. Therefore, in the current study, an attempt also has been made to analyze and forecast by using a combination of ARIMA and ANN models. By comparing three ARIMA, ANN and hybrid approaches, we can achieve the most accurate method for predicting the demand for blood groups.

In the hybrid model used in this study, which is adapted from Zhang's study^16^, the task is to model by successively applying ARIMA and ANN approaches. Based on this point of view, the observations consist of two components^16,22^. The linear component which can be identified and modeled using ARIMA, and the non-linear complex component that cannot be identified and modeled using regular seasonality in ARIMA models. Therefore, in the modeling of the nonlinear component, the neural network method is proposed, which has previously been proven to be more effective in predicting complex nonlinear relationships^25^. In the other words.

$$observation=linear \; component+nonlinear \; component+residual$$

Thus in the hybrid approach, it is suggested to first fit the ARIMA model to the data and then use the ANN algorithm on the residuals of fitted ARIMA, so that if there are any nonlinear components, this value can also be identified and removed from observations to obtain white noise residuals. Therefore, the prediction of the hybrid model will be:

$$predicted \; value=ARIMA \; prediction+ANN \; prediction$$

### Seasonality

According to the used data (monthly blood demand data for 8 years (96 monthly points)), there was a possibility of a periodic effects with fixed period length, known as seasonal effects in the data. Moreover assessing the data pattern (before differencing) reinforced this idea. “m <-decompose(data); plot(m);” command in “forecast” package was used for checking the seasonality in data plot. According to the supplementary Fig. 1, presence of seasonality pattern was confirmed in all blood groups data (except group B−).

To estimates the number of seasonal differences, Canova-Hansen^27^ and Osborn-Chui-Smith-Birchenhall^28^ tests were used. For this aim we used "nsdiffs” function in forecast package. It uses seasonal unit root tests to specify the seasonal differences number required for a special time series to be made stationary.

### Model validation and statistical comparisons

To validate and comprise two ARIMA and ANN models, MSE and mean absolute error (MAE) criteria were used. So that data were divied into two parts. The data of first 84 months were choosed as training and last 12 months as testing. Finally, ARIMA and ANN estimates for last 12 months were compaired to actual data. Exell 2016, R3.6.3 were used for data analysis. The “nnfor” and “forecast” were the main packages which used for modelling. Moreover, other packages including “openxlsx” and "nonlinearTseries" were respectively applied for importing data and other tests and analysis.

It should be noted that the study protocol of the current research was approved by the Ethics Committee of Shiraz University of Medical Sciences under code IR.SUMS.REC.1398.1275.

### Ethics approval

The project was found to be in accordance to the ethical principles and the national norms and standards for conducting medical research. The study protocol of the current research was approved by the Ethics Committee of Shiraz University of Medical Sciences under code IR.SUMS.REC.1398.1275.

### Informed consent

In the current research, aggregate monthly data on the demand of Shiraz hospitals and medical centers for 8 blood groups (A+, A−, B+, B−, AB+, AB−, O+, O−) during 2012–2019 were gotten from Shiraz branch of Iranian Blood Transfusion Organization. So informed consent was not applicable.

## Results

### ARIMA estimates

**Figure 2:**
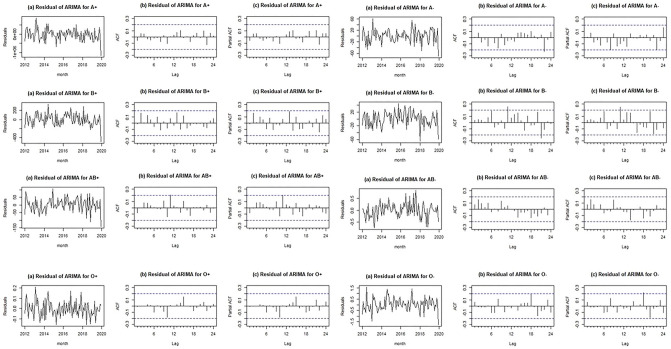
Residuals, ACF and PACF charts for ARIMA.

Graphs of residuals (part a), AFC (part b), and PACF (part c) for eight blood groups (Fig. 2) showed that there are not significant dispersion, autocorrelation, and partial autocorrelation for all blood groups data.

According to the AIC, AICc, and BIC indicators, proposed ARIMA model for eight blood groups are shown in the Table 1.

**Table 1:** Metrics for the selected ARIMA models.

Blood group	Model	AIC	AICc	BIC
A+	(0,0,0)(1,0,1)	2719.15	2719.59	2729.41
A−	(0,0,0)(1,0,0)	878.26	878.53	885.96
B+	(0,0,0)(1,0,0)	1218.28	1218.54	1225.98
B−	(1,0,1)	865.57	866.01	875.83
AB+	(0,0,0)(2,0,0)	1011.6	1012.04	1021.86
AB−	(0,0,0)(2,0,0)	53.57	50.57	55.57
O+	(2,0,0)(1,0,1)	− 204.47	− 203.53	− 189.08
O−	(0,0,0)(2,0,0)	176.39	176.83	186.64

Moreover, based on White neural network, McLeod–Li, Box–Ljung, and Shapiro–Wilk tests (P-value > 0.01) models residuals for almost all blood groups were stationary, homoscedasticite, Independence, and normal (Table 2).

**Table 2:** Nonlinearity, heteroscedasticity, independence, and normality tests for ARIMA residuals.

Blood groups	White neural network (X^2^,df,p-value)	McLeod–Li (df,p-value)	Box–Ljung (X^2^,df,p-value)	Shapiro–Wilk (W,p-value)
A+	(3.8, 2, 0.15)	(2, 0.98)	(0.37, 1, 0.54)	(0.98, 0.26)
A−	(2.6, 2, 0.26)	(2, 0.99)	(0.0014,1,0.97)	(0.99, 0.67)
B+	(0.28, 2, 0.87)	(2, 0.99)	(0.016, 1, 0.9)	(0.98, 0.08)
B−	(3.7, 2, 0.156)	(2, 0.98)	(0.11, 1, 0.73)	(0.98, 0.3)
AB+	(0.11, 2, 0.94)	(2, 0.96)	(0.68, 1, 0.4)	(0.99, 0.7)
AB−	(0.06, 2, 0.97)	(2, 0.78)	(0.5, 1, 0.47)	(0.99, 0.7)
O+	(3.8, 2, 0.15)	(2, 0.73)	(0.002,1, 0.96)	(0.98, 0.54)
O−	(10.5, 2, 0.005)	(2, 0.98)	(0.4, 1, 0.52)	(0.98, 0.29)

### ANN estimates

Selected ANN structure for each blood groups are shown in the Table 3. Final models are selected based on minimum value of MSE. Error values are specified by changing the number of neurons in the hidden layers, lags, and Reps. There are different lags for different blood groups. Lags indicate the prediction steps which are used to estimate next-step prediction e.g. lag 24 for blood group A+ means that we applied 24 first observations to predict 25th step in the network. Moreover, reps show that for each blood group 100 neural networks with mentioned layers and neurons were constructed and the final result was the average prediction of different networks, known as ensemble forecasting. The full information for each blood group were presented in Table 3.

**Table 3:** The best neural network models for blood groups according to the MSE criteria.

	Blood groups							
	A+	A−	B+	B−	AB+	AB−	O+	O−
Layers (nodes)	(20,12,5)	(10,3)	(20,5)	(8,5)	(12,10,2)	(5)	(5)	(12,5)
Lags	24	24	20	24	12	12	24	24
Reps	100	100	100	100	100	100	100	100

**Figure 3:**
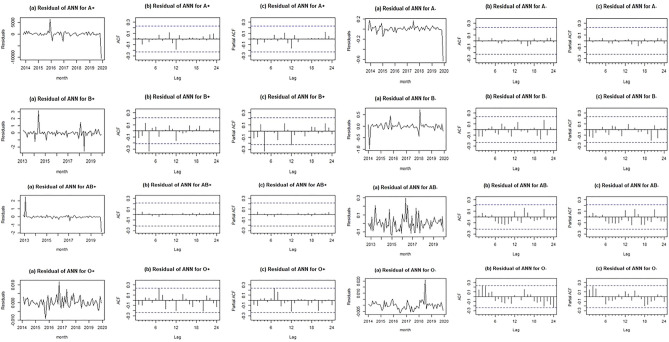
Residuals, ACF and PACF charts for ANN.

According to the graphs of residuals (part a), AFC (part b), and PACF (part c) (Fig. 3), there are not significant dispersion, autocorrelation, and partial autocorrelation for all blood groups data. Moreover, residuals assessment confirms the sufficiency of fitted model and indicate that the selected algorithm is consistent with the blood demand time series data.

### Hybrid model estimates

In hybrid approach, after fitting the ARIMA model of the orders reported in Table 1, the ANN is fitted on the residuals. Table 4 shows the architecture of the ANN fitted on the residuals. The number of hidden layers, neurons in hidden layers, Lags and Reps are selected in order to minimize the MSE of the network.

**Table 4:** The best hybrid models for blood groups according to the MSE criteria.

	Blood groups							
	A+	A−	B+	B−	AB+	AB−	O+	O−
Layers (nodes)	(5)	(5)	(5)	(5)	(5)	(5)	(5)	(5)
Lags	12	12	12	12	12	12	12	12
Reps	100	100	100	100	100	100	100	100

**Figure 4:**
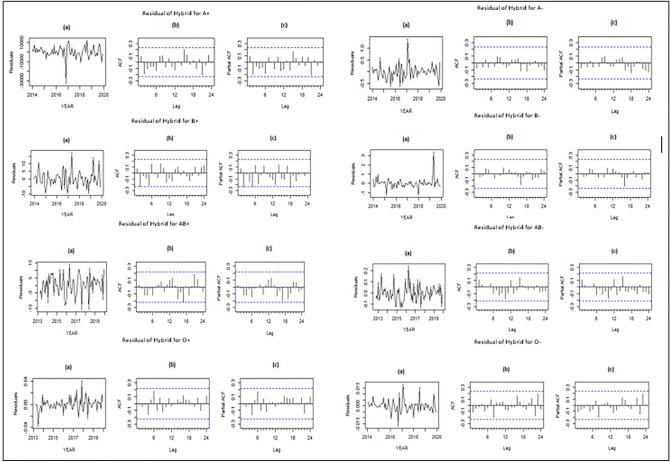
Residuals, ACF and PACF charts for hybrid model.

The plots of the final residuals of the hybrid model are depicted in the Fig. 4. As can be seen, for all blood groups, the residuals are randomly scattered around zero without any trend. According to the ACF and PACF charts, all the peaks are within the confidence limits, which shows that there is no correlation or autocorrelation in the residuals. This indicates the adequacy of the fitted model.

### Comparidopn of ARMIA, ANN and hybrid models

In order to facilitate the comparison of the modles with each other, the ratio of ANN and hybrid error (MAE and MSE) to ARIMA error is provided (Table 5). According to the error ratios (MAE values), it can be claimed that for all blood groups except O+ and O−, the ARIMA method performed better than the other method and the neural network algorithm and the hybrid model performed almost the same. In addition, for O+ and O− blood groups, the ANN had the lowest absolute error in fitting and prediction. Based on MSE, the results are also similar to the mentioned findings thus the prediction error of the ARIMA was less or equal to the ANN and the hybrid model for almost all blood groups except O+ and O−. In addition, for the O+ and O− groups, the neural network algorithm has the lowest mean square error in fitting and prediction.

**Table 5:** Comparison of ARIMA, ANN and hybrid models based on MAE and MSE criteria.

Blood groups	ARIMA		ANN		Hybrid		MAE		MSE	
	MAE	MSE	MAE	MSE	MAE	MSE	$\frac{ANN}{ARIMA}$	$\frac{Hybrid}{ARIMA}$	$\frac{ANN}{ARIMA}$	$\frac{Hybrid}{ARIMA}$
A+	8.81E−02	5.37E+04	9.40E−02	4.88E+04	9.32E−02	5.77E+04	1.07	1.06	0.91	1.07
A−	1.45E−01	9.92E+02	1.65E−01	1.41E+03	1.55E−01	2.00E+03	1.14	1.07	1.42	2.02
B+	7.95E−02	3.22E+04	1.00E−01	3.83E+04	8.53E−02	2.88E+04	1.26	1.07	1.19	0.89
B−	1.32E−01	6.26E+02	1.67E−01	1.02E+03	1.51E−01	8.81E+02	1.27	1.14	1.63	1.41
AB+	8.93E−02	3.05E+03	1.23E−01	5.99E+03	1.24E−01	5.25E+03	1.38	1.39	1.96	1.72
AB−	8.01E−02	3.38E+01	3.14E−01	3.57E+02	9.26E−02	4.78E+01	3.92	1.16	10.56	1.41
O+	5.72E−02	6.67E+04	3.18E−02	4.91E+04	5.78E−02	5.66E+04	0.56	1.01	0.74	0.85
O−	1.32E−01	2.05E+03	8.90E−02	1.11E+03	1.45E−01	3.21E+03	0.67	1.10	0.54	1.57

**Figure 5:**
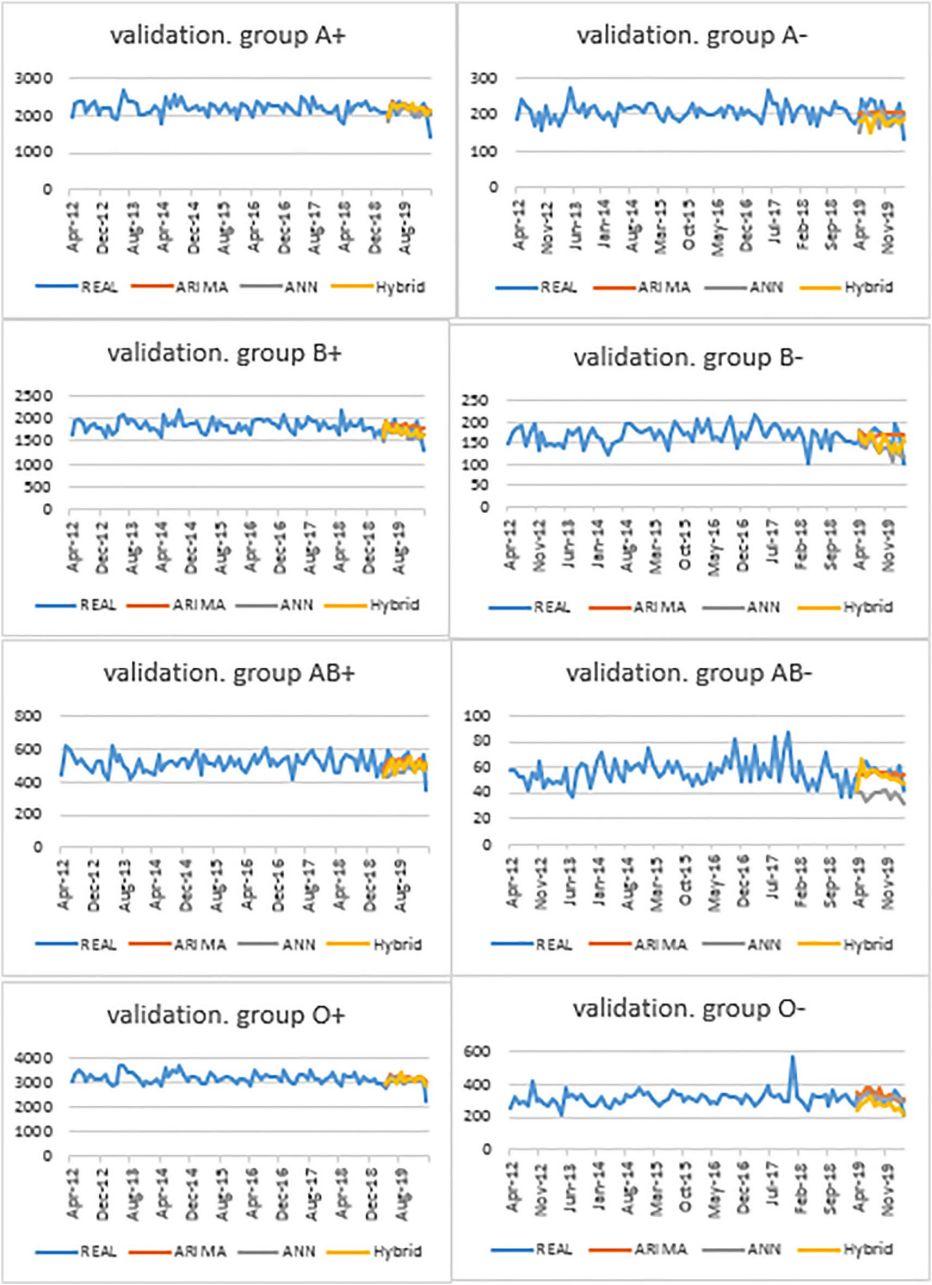
Comparison of prediction of ARIMA, ANN and hybrid models with actual data.

Moreover, comparison of ARIMA, ANN and hybrid models validation graphs indicated that, for most blood groups, ARIMA had closer prediction to actual data (Fig. 5).

### Overall demand trend

**Figure 6:**
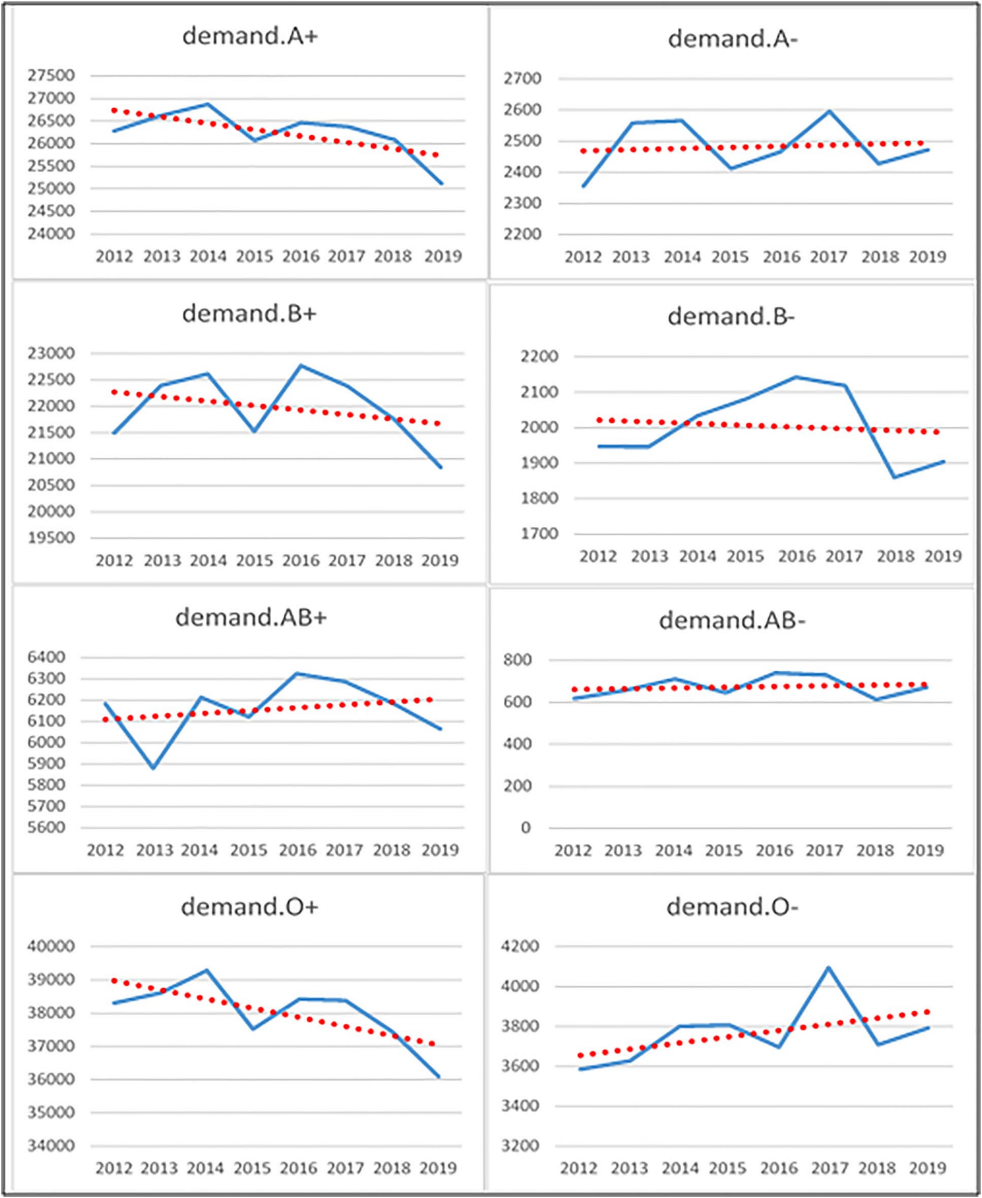
Trend of demand for blood by different blood groups.

**Figure 7:**
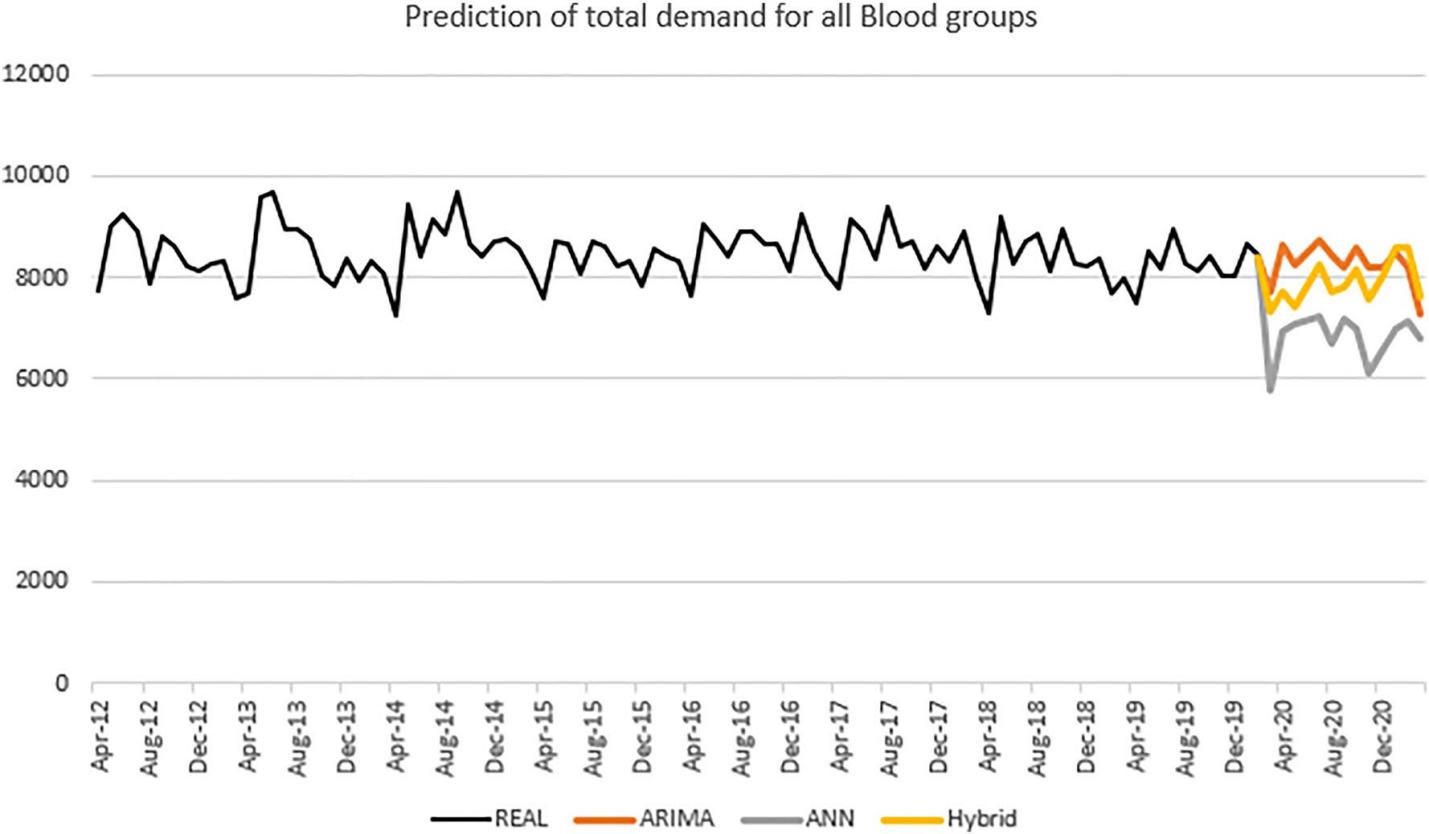
Forecasts of overall blood demand using ARIMA, ANN and hybrid models.

Trend of demand between 2012 to 2019 showed that there are decreasing trend for positive blood groups and increasing trend for negative blood groups (Fig. 6). Prediction of overall blood demand according to ARIMA, ANN and hybrid models are shown in the Fig. 7.

## Discussion

Access to fresh blood has always been a main medical issue to keep people in need of blood alive. Proper policy-making in this area requires accurate forecasting of blood demand. The aim of this study was to predict blood demand by blood groups during 1 year in Shiraz using ARIMA, ANN and a hybrid approaches.

The findings were discussed in two sections, the first of which compared demand forecasts for different blood groups based on the three approaches, and the second dealt with the overall demand trend over the years of study.

### Performance of models in predicting blood demand

The findings of this study showed that the ARIMA model had better performance than ANN and hybrid model in all blood groups except for O+ and O− in which the ANN have better prediction.

The studies conducted on blood demand prediction reported different results in terms of the accuracy of different methods in predicting the demand. Turkulainen in Finland examined the performance of statistical models for predicting short-term and long-term blood demand. Based on various error indices, the best models differed in different blood groups, but ARIMAX was mostly introduced as the best^17^.

Fanoodi et al. in Iran and Khaldi et al. in Morocco conducted similar research and concluded that the ANN method was more efficient than ARIMA for predicting blood demand^14,29^.

Tanyavutti and Tanlamai in Thailand used Box-Jenkin, ARIMAX, and Holt Winter methods and predicted blood demand with the least absolute error. The ARIMAX method had fewer errors in most cases^30^.

In the United States, Fortsch and Khapalova used daily data and six different methods to predict blood demand. They finally introduced the Box-Jenkins, ARMA, method as the best model for predicting the total demand and the demand for all blood groups except A−^18^.

Pereira used the following three methods to predict the demand for red blood cells in Spain: ARIMA, ETS, and ANN. The results showed that in 1-year time horizons, RBC demand forecasting with ARIMA or ETS methods performed better, but in longer time horizons, ETS had better performance than other forecasting methods^19^.

Filho et al. conducted a study in Brazil to predict the demand for distribution of blood components in order to minimize blood product wastage. Instead of using the traditional Moving-Average method with 1-week lag, they suggested the Box-Jenkins method (BJ) and approved the Seasonal ARIMA (SARIMA) model^31^.

Jiang forecasted the emergency blood demand after earthquake using an adaptive evolutionary support vector regression. It said that their model provides a favorable accuracy than ANN and other traditional models^32^.

In another study, a multivariate time-series blood supply and demand prediction model was used for resilient supply chain management during COVID-19 pandemic. They proposed a LSTM, a recurrent neural network, for blood forecasting^33^.

In studies other than those conducted to predict blood demand, the results of different models are also different. For example Khan et al. used a hybrid ANN-ARIMA model for meteorological drought forecasting. They conducted that ARIMA-ANN hybrid model performs better than the two separate models^34^. Perone compared the ARIMA, ETS, NNAR, TBATS and hybrid models to predict the second wave of COVID-19 hospitalizations in Italy. Finally, the hybrid models were suggested^35^. On the other hands, in a study for forecasting milk production in South Asian countries, comparison of mean absolute percentage errors showed that ARIMA had lower error that Holt’s linear model^36^.

In the above some studies with the same model as our study, the results are opposite to the results of our study in terms of model error. In the regard to the differences between various models, it can be said that the small sample size in the current study has led to the small sample size in the train data set, and as a result, fitting the ANN in a small set has led to its over-fitting. The over-fitting of the neural network in both ANN and hybrid model has led to an increase in the error in the validation part. Therefore, as a suggestion for future studies with large data sets, it is possible to examine the over-fitting of the neural network and compare the models in the entire data set as well as a random subset of the same data set in order to be able to test the hypothesis of more over-fitting of the neural network in the data set with a smaller sample size. Moreover, differences in the performance of various models used in different studies could be due to the reasons such as different forecast horizons, daily/monthly/annual data, different sample sizes, types of demand variables and the transformation applied on them, different random shocks, and finally different blood demand behaviors in communities during different periods.

### Blood demand behavior

In the present study, the demand for four blood groups (mostly negative groups) was increasing and the demand for other four blood groups (mostly positive ones) was decreasing. Both ARIMA, ANN and hybrid models predicted an almost downward trend for the total blood demand. Previous studies had reported different behaviors for blood demand in the studied population.

Laur´en et al. showed that despite the increasing population as well as the increasing demand for health care, RBC consumption had decreased in Finland. The use of advanced surgical techniques and intraoperative cell salvage, increased public awareness, the lack of using synthetic colloids, more limited administration of intravenous fluids, minimizing iatrogenic haemodilution, reduced birth rate, and emphasis on the use of single-unit injections were claimed to be the reasons for such reduction^37^.

Borkent-Raven et al. in the Netherlands used two models for the RBC demand forecasting. Accordingly, they predicted an increase of 23% in RBC demand using the demographic-based model, and a decrease of 8% using the model based on demographic changes and RBC consumption trend. To justify this result, they stated that in the second model, the effect of increased aging trend on the demand for blood and blood products was remarkably modified by the factors such as the optimal use of blood^11^.

Sasongko interviewed some experts and reviewed previous studies, based on which four scenarios were predicted for blood demand in the Netherlands: 17% upward trend, 45% downward trend, 12% stable downward trend, and 7% stability. They provided some reasons for each scenario. The reasons for the blood demand decrease scenario included gene therapy, better surgical techniques, precision medicine, higher awareness of the side effects of blood transfusions, innovations in hemoglobinopathy, and reduced use of blood in cesarean sections and other surgeries. On the other hand, factors such as population aging, high burden of non-communicable diseases (NCDs), the use of RBC injections instead of crystalloids and colloids to treat hemorrhagic shocks, and the support for intensive care unit (ICU) patients were mentioned as the causes of increased blood demand^38^.

In their studies, Drackley et al. in Canada, Seifried et al. in Germany, and Volken et al. in Switzerland referred to the relationship between the elderly population and the increased blood demand, resulting in an imbalance in blood supply and demand^39–41^.

In the present study, the general trend of demand was slightly downward. Advances in surgical techniques, fetal screening, reduction of accidents leading to heavy bleeding, and the modified pattern of blood request for surgeries appeared to have been effective in reducing the demand trend.

### Limitations

Although this study was conducted in Shiraz city where there was huge blood demand for a large population, it cannot be generalized to the whole country. In addition, the present study used the data of 96 months to compare and forecast blood demand. However, a longer time period would certainly provide more accurate estimates. Another issue is that residual assessment and other goodness of fit measures of models which used in the current study confirmed their suitability. However other models such as Long Short Term Memory (LSTM) networks, Support Vector Regression (SVR), Gate-variants of gated recurrent unit (GRU) neural networks are also proposed by researchers^42–44^ may have better estimates. Using of these models for prediction of blood demand can be applied in future studies.

## Conclusion

The results of this study indicated that with the available data and based on the MAE and MSE error criteria, the ARIMA model made a more accurate forecast of blood demand (in most groups) for a 12-month horizon compared to the ANN and hybrid model. The demand trend was also downward in most positive groups (A+, AB+, O+) and upward in most negative ones (A−, B−, O−).

## Supplementary Information

Supplementary Figure S1.

## Data Availability

The datasets used and analyzed during the current study are available from the corresponding author on reasonable request.

## Abbreviations

ACF: Autocorrelation
AIC: Akaike information criterion
AICc: Corrected Akaike information criterion
ANN: Artificial neural network
ARIMA: Auto regressive integrated moving average
ARIMAX: ARIMA with exogenous regressors
BIC: Minimum Bayesian information criterion
BJ: Box–Jenkins
ETS: Exponential smoothing models
GRU: Gate-variants of gated recurrent unit
ICU: Intensive care unit
LSTM: Long short term memory
MAE: Mean absolute error
MLP: Multi-layer perceptron
MSE: Mean square error
NCDs: Non-communicable diseases
PACF: Partial autocorrelation
RBC: Red blood cell
SARIMA: Seasonal ARIMA
SVR: Support vector regression
WHO: World Health Organization
