# Supplementary figures and images for: Forecasting blood demand for different blood groups in Shiraz using auto regressive integrated moving average (ARIMA) and artificial neural network (ANN) and a hybrid approaches
Source: Sci Rep. 2022 Dec 20;12:22031. doi: 10.1038/s41598-022-26461-y (PMC9767396; doi:10.1038/s41598-022-26461-y)

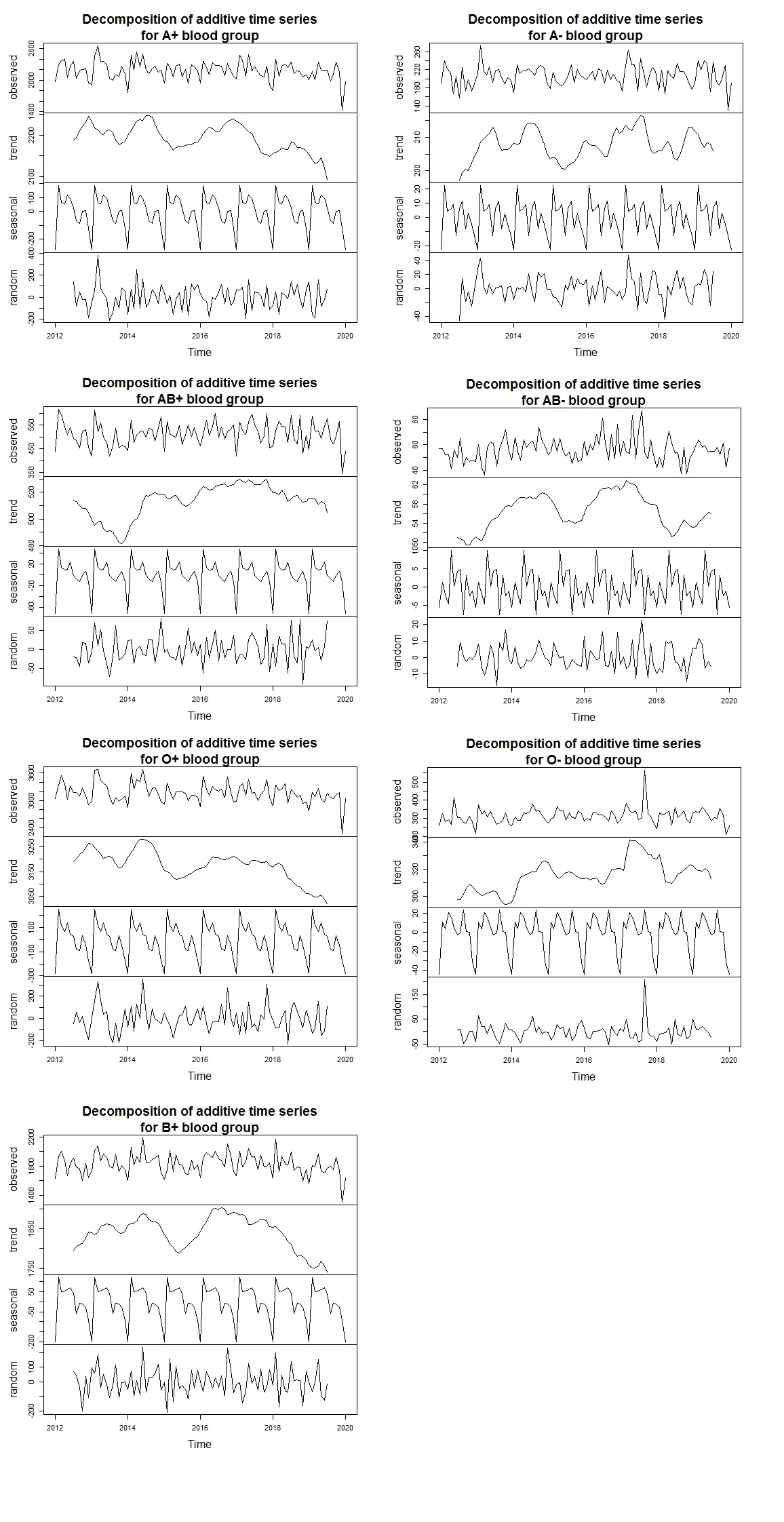

Supplement: Supplementary file 1 — Supplementary Figure S1. [file 41598_2022_26461_MOESM1_ESM.jpg]
